# Inferring functional traits in a deep-sea wood-boring bivalve using dynamic energy budget theory

**DOI:** 10.1038/s41598-021-02243-w

**Published:** 2021-11-22

**Authors:** S. M. Gaudron, S. Lefebvre, G. M. Marques

**Affiliations:** 1 UMR 8187, Laboratoire d’Océanologie et de Géosciences (LOG), Université de Lille, ULCO, CNRS, 59000 Lille, France; 2 Sorbonne Université, UFR 927, 75005 Paris, France; 3 MARETEC—Marine, Environment & Technology Center, LARSyS, Instituto Superior Técnico, Universidade de Lisboa, Lisboa, Portugal

**Keywords:** Ecological modelling, Ecophysiology, Evolutionary ecology, Ecology, Ecology, Ecological modelling, Ecophysiology, Evolutionary ecology, Ocean sciences, Marine biology

## Abstract

For species in the deep sea, there is a knowledge gap related to their functional traits at all stages of their life cycles. Dynamic energy budget (DEB) theory has been proven to be an efficient framework for estimating functional traits throughout a life cycle using simulation modelling. An abj-DEB model, which compared with the standard DEB model includes an extra juvenile stage between the embryo and the usual juvenile stages, has been successfully implemented for the deep-sea Atlantic woodeater *Xylonora atlantica*. Most of the core and primary parameter values of the model were in the range of those found for shallow marine bivalve species; however, in comparison to shallow marine bivalves, *X. atlantica* required less energy conductance and energy to reach the puberty stage for the same range of body sizes, and its maximum reserve capacity was higher. Consequently, its size at first reproduction was small, and better survival under starvation conditions was expected. A series of functional traits were simulated according to different scenarios of food density and temperature. The results showed a weak cumulative number of oocytes, a low growth rate and a small maximum body size but an extended pelagic larval duration under deep-sea environmental conditions. Moreover, DEB modelling helped explain that some male *X. atlantica* individuals remain dwarfs while still reproducing by changing their energy allocation during their ontogenetic development in favour of reproduction. The estimation of functional traits using DEB modelling will be useful in further deep-sea studies on the connectivity and resilience of populations.

## Introduction

The deep sea has been recognized as a frontier of science and discovery, and advancing fundamental biological and ecological knowledge is urgently necessary in the next decade^1^. A blueprint on the accomplishments needed for the next decade was established by an international workgroup of experts in deep-sea biology and ecology, and some of the priority measurements needed were feeding regime, growth rate, longevity, fecundity, size at puberty, maximum body size and pelagic larval duration for benthic and pelagic deep-sea macro- and megafauna^2^. This fundamental knowledge is necessary for understanding the connectivity and resilience of species populations and trophic network interactions in the ocean. The deep sea, which is considered below 200 m to abyssal plains (8000 m deep), encompasses ecosystems^3^ such as wood-fall ecosystems that are patchy and ephemeral habitats (occur for only a few years)^4^. During extreme weather events, numerous wooden logs are transported offshore by oceanographic processes and, after being saturated with seawater, sink to the deep sea^5^. Sunken wood brings considerable carbon flux to a food-limited seafloor. Wood-fall communities are composed primarily of endemic wood species and sulfide symbiotrophic species^6^. Among them are the mollusc wood-boring bivalves of the family Xylophagaidae, of which numerous species inhabit several ocean basins below 200 m^7–9^. In shallow water, a different family of wood-boring bivalves occurs, the Teredinidae family (shipworms)^10^. This family is often seen as a pest^11^, boring into fishing gear, ships and wooden equipment, with a distribution ranging from intertidal to 100 m deep. Therefore, these two families (Teredinidae and Xylophagaidae) do not share the same bathymetrical niche within the ocean, although both borers are xylotrepetic and xylotrophic marine invertebrates^12, 13^. In fact, these marine bivalves are considered symbiotic species in that they host bacterial endosymbionts within their gills. These bacteria produce cellulolytic enzymes that help degrade the wood debris stored within the wood-borer’s caecum, and at the same time, the symbiont is able to fix nitrogen to compensate for the poor nitrogen content in wood^13–15^.

Dynamic energy budget (DEB) theory^16^ enables a trait-based bioenergetic and mechanistic approach to evaluate functional traits using simulation modelling^17–19^. A DEB model quantifies the energy allocation to reproduction (or acquisition of complexity) and growth of a species according to environmental conditions such as temperature and food availability at any stage of its life cycle^16^. Life-history traits (zerovariate data such as age at puberty) and observational or experimental data associating a dependent variable with an independent variable (univariate data such as length over time) are necessary to parameterize such a model. *Xylophaga atlantica,* renamed recently *Xylonora atlantica*^20^ known as the Atlantic woodeater, lives from 100 to 7000 m in depth^8^ and belongs to the Xylophagaidae family. This species has been reared in the laboratory, providing some information on traits at the larval to juvenile stages^21^. In situ colonization experiments were carried out in the Atlantic Ocean at a depth of 100 m to collect *X. atlantica* at different time periods, providing further data on growth^22^. More recently, colonization experiments using a novel conceptual colonization device named ‘CHEMECOLI’^23^ deployed at 2300 m in a hydrothermal vent ecosystem on the mid-Atlantic ridge permitted the collection of very preserved specimens of *X. atlantica*. In the present study, biometric measurements were carried out on the wet weight and shell height of *X. atlantica,* allowing the shape of the species (parameter needed for the DEB model) to be calculated. Moreover, a recent histological study based on specimens collected from these CHEMECOLI provided new data on their reproduction^24^. These zerovariate and univariate data helped calibrate the DEB model. The DEB model was used to infer missing functional traits such as fecundity during lifespan, growth rate, lifespan and pelagic larval duration at deep sea.

There is a common belief that the metabolism of deep-sea species is much lower than that of their shallower-living relatives, with several factors explaining this observation, such as a decrease in temperature and food availability, an increase in hydrostatic pressure, and a decline in oxygen^25, 26^. Up to a depth limit (~ 2000 m), temperature seems to covary with hydrostatic pressure and has been considered a confounding factor^26^. A decrease in metabolism with depth is mostly due to the direct effect of temperature rather than hydrostatic pressure^25^. In the present study, temperature was used as a forcing variable within the DEB model, where a low temperature was a proxy of deep wood-fall habitats, and a higher temperature was a proxy of shallow wood-fall habitats. The second forcing variable used within the DEB model was food density. In the deep sea, the flux of carbon from the surface controls metabolic rates, and the flux decreases approaching the abyssal depth^27, 28^*,* except in chemosynthetic ecosystems such as hydrothermal vents, where energy availability is much higher, resulting in some vent benthic species having metabolic rates similar to those of their shallower counterparts (e.g., brachyuran crabs)^29^. Xylophagaid wood borers are supposed to be strictly xylophages, but one cannot discard individuals who filter-feed on particle organic matter (POM) produced in vent ecosystems, as hypothesized recently for some species of the xylophagaid clade^30^. Plasticity in reproduction has been observed in *Xylonora atlantica,* where some tiny individuals sexed as males and called dwarf males were recovered on the dorsal side of some female *X. atlantica* in the colonization device CHEMECOLI^23, 24^. The dwarf males were not equipped to bore wood as the remaining ‘normal’ *X. atlantica* males and females could, but they were mature, carrying mature sperm and filter feeding^24^. Questions remain regarding how these tiny specimens of *X. atlantica* were able to reproduce having the size of young juveniles.

To explore different functional traits (von Bertalanffy growth, fecundity during lifespan, maximal body size, age at puberty, and pelagic larval duration) to infer *X. atlantica*’s ecology, an abj-DEB model was developed for this species and for the first time for a benthic deep-sea species. The core and primary parameters of the DEB model of *X. atlantica* were compared to those of a shallower woodeater (*Teredo navalis*) and to those of their closest marine taxa within the bivalve clade. In addition, using this novel bioenergetic model, a new hypothesis was presented on how the dwarf male stage in *X. atlantica* is possible.

## Results

### Parameterization of an abj-DEB model for *Xylonora atlantica*

**Figure 1:**
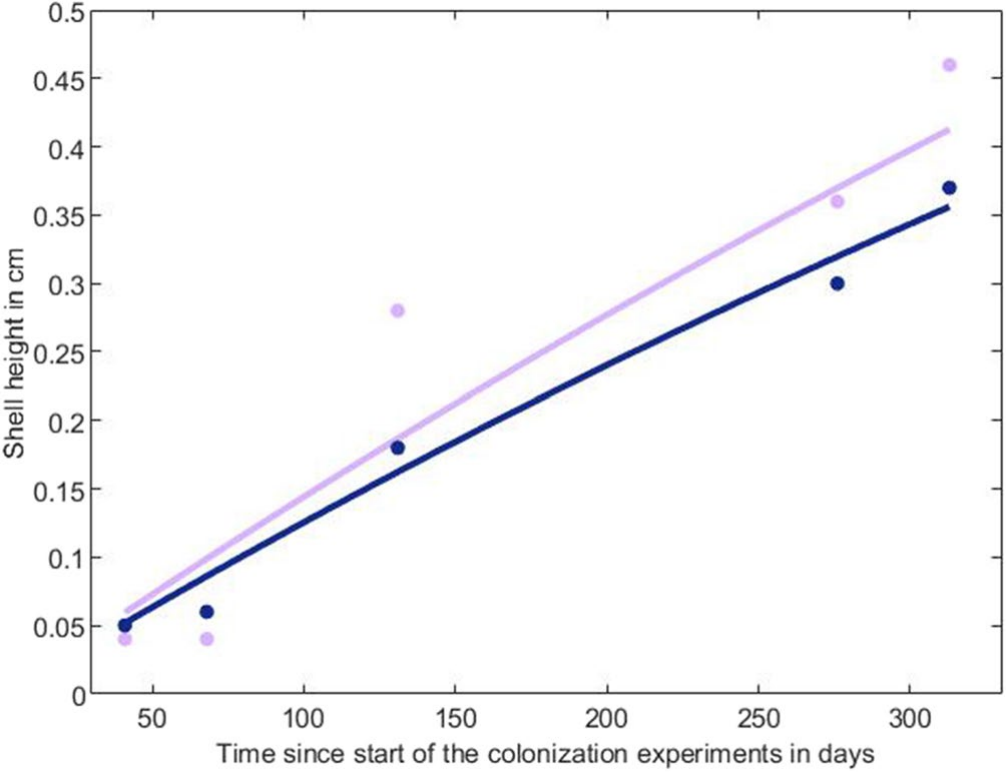
Observed (dots) and predicted (lines) data on the growth of *Xylonora atlantica* in shell height before and after using the abj-DEB model. Data from the pine substrate are in purple, and those from the oak substrate are in blue. The observed data were obtained from in situ colonization experiments at a depth of 100 m^22^ and were the mean values of 8 to 174 individuals depending on the sampling period or wood type. The relative error of the data predicted (RE) for the pine substrate (purple) was 0.19, and the RE for the oak substrate (blue) was 0.08.

For the parameter estimation, the completeness of real data^31^ within the abj-DEB model developed for *X. atlantica* was 2.6 using the dataset of online Supplementary Table S2. This value of 2.6 is in the mean of the values found in the ‘Add_my_pet’ database, highlighting the good quality and availability of the data. The goodness of fit^31^ resulted in a mean relative error (MRE) of 0.148 and a symmetric mean squared error (SMSE) of 0.141, demonstrating a good match between observations and predictions. The model has been validated and published online within the ‘Add_my_pet’ database (https://www.bio.vu.nl/thb/deb/deblab/add_my_pet/entries_web/Xylonora_atlantica/Xylonora_atlantica_res.html). Out of the 12 zerovariate data (Table 1), most of the predicted values (12) were close to the observed values (0.004 ≤ RE ≤ 0.38). For the univariate data, the relative error (RE) varied from 0.08 to 0.19 for the two in situ colonization experiments. The scaled functional response (*f*) is a limiting function that varies between 0 (starvation) and 1 (satiety) and depends on food availability. *f* was fixed at 1 for the pine substrate, giving a smaller *f* for the oak substrate that was left free within the calibration procedure of the abj-DEB model and estimated at 0.79 (Fig. 1). This result highlights that the food availability provided by the pine substrate was greater than that provided by the oak substrate, resulting in better growth of *X. atlantica* on pine than on oak logs (Fig. 1). The values of the shape coefficient were 0.629 for the larval stage (*δ*_*ME*_) and 0.599 for the juvenile/adult stage (*δ*_*M*_) (Supplementary Table S3 and Fig. S2 online).

**Table 1:** Summary of the observed zerovariate data (life-history traits) and those predicted by the abj-DEB model of *Xylonora atlantica.*

Data	Symbol	Value observed	Value predicted (RE)	Unit
Age at hatching (trochophore)	*a*_*h*_	2	2.48 (0.24)	d
Age at birth (first feeding)	*a*_*b*_	25	15.49 (0.38)	d
Time since birth to metamorphosis	*t*_*j*_	28.4	28.75 (0.07)	d
Time since birth to puberty	*t*_*p*_	108	125.2 (0.16)	d
Lifespan	*a*_*m*_	547	544.5 (0.005)	d
Height of the trochophore (hatching)	*Lw*_*h*_	0.004	0.0032 (0.19)	cm
Shell height of the umboveliger (birth)	*Lw*_*b*_	0.0135	0.0167 (0.24)	cm
Shell height at the metamorphosis	*Lw*_*j*_	0.035	0.047 (0.34)	cm
Shell height at puberty (sexual maturation)	*Lw*_*p*_	0.2	0.197 (0.02)	cm
Maximum shell height	*Lw*_*i*_	1.5	1.6 (0.06)	cm
Wet weight at puberty	*Ww*_*p*_	0.004	0.0038 (0.06)	g
Maximum reproduction rate	*R*_*i*_	10.85	10.81 (0.004)	oocytes.d^−1^

*RE* relative error; *d* days.

### Interspecific comparisons of core and primary parameter values of *Xylonora atlantica*, *Teredo navalis* and other shallow marine bivalve species

**Figure 2:**
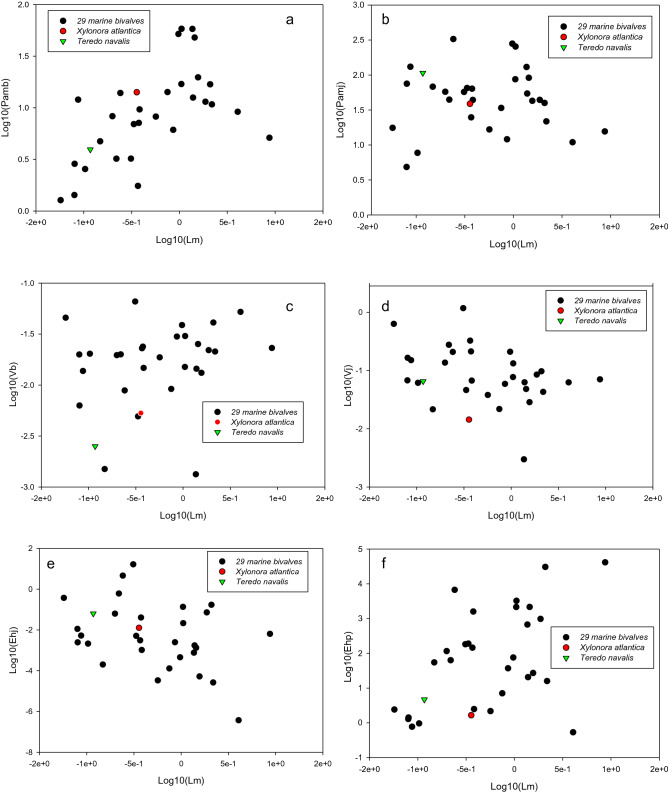
Interspecific comparison of abj-DEB parameter values of 29 marine bivalves with those of the deep-sea wood borer *Xylonora atlantica* and the shallow wood borer *Teredo navalis*, scaled to body size (Lm: maximum structural length) (**a**) Pamb*,* maximum assimilation rate at birth ${\left\{{\dot{p}}_{Am}\right\}}_{b}$), (**b**) Pamj, maximum assimilation rate after metamorphosis ${\left\{{\dot{p}}_{Am}\right\}}_{j}$; (**c**) Vb*,* energy conductance at birth ${\dot{v}}_{b}$; (**d**) Vj*,* energy conductance after metamorphosis ${\dot{v}}_{j};$ (**e**) Ehj*,* energy threshold after metamorphosis ${E}_{H}^{j}$, and (**f**) Ehp*,* energy threshold at puberty ${E}_{H}^{p}$. The black circles are the values for the 29 marine bivalves for which an abj-DEB model was applied. The red circles are the DEB parameter values of *X. atlantica.* The green triangles are the DEB parameter values of *T. navalis*.

The $\kappa$ value (0.73) and maximum assimilation rate at birth ${\left\{{\dot{p}}_{Am}\right\}}_{b}$ of *X. atlantica* were in the same range as those of other marine bivalves (Table 2; Fig. 2a); however, the $\kappa$ for *T. navalis* was higher (0.98) and ${\left\{{\dot{p}}_{Am}\right\}}_{b}$ was very low (Table 2). The maximum assimilation rate after metamorphosis ${\left\{{\dot{p}}_{Am}\right\}}_{j}={\left\{{\dot{p}}_{Am}\right\}}_{b} {s}_{M}$ of *X. atlantica* was twofold lower than that of other marine bivalves (Table 2) and threefold lower than that of the shallow wood borer *T. navalis*. However, when the value of ${\left\{{\dot{p}}_{Am}\right\}}_{j}$ was compared to those of the same range of body sizes (Fig. 2b), the value was in the same order. Energy conductance values ($\dot{v}$) of both wood-boring bivalves were lower at birth (${\dot{v}}_{b}$) and after metamorphosis (${\dot{v}}_{j}={\dot{v}}_{b}{s}_{M}$) compared to these values of other marine bivalve species, with values that were fourfold lower at birth and more than tenfold lower after metamorphosis for *X. atlantica* and only twofold lower for *T. navalis* (Table 2). Once body size was scaled, as seen in Fig. 2c,d, ${\dot{v}}_{b}$ and ${\dot{v}}_{j}$ of *X. atlantica* were still much lower than those of other marine bivalves of the same body size, but only ${\dot{v}}_{b}$ was still lower for *T. navalis* (Fig. 2c,d). The maximum reserve capacity $\left[{\mathrm{E}}_{m}\right]$ of *X. atlantica* was twofold higher than that of *T. navalis* and fourfold higher than those of the remaining marine bivalves (Table 2). For *X. atlantica*, the output of the abj-DEB model had a time of starvation (‘t starve’) equal to 447 days at 4 °C. The volume-specific cost for structure $\left[{\mathrm{E}}_{G}\right]$ was nearly equal among all marine bivalve species (Table 2). The volume-specific somatic maintenance $\left[{\dot{p}}_{M}\right]$ and the somatic maintenance rate coefficient (${\dot{k}}_{M}$) of the deep-sea xylophagaid were higher than those of the other marine bivalve species (Table 2). The maximal structural length (*L*_*m*_) of *X. atlantica* was lower than the mean values of the 29 marine bivalve species (Table 2), but in Fig. 2, the *L*_*m*_ of *X. atlantica* appeared in the middle range of the values. Less energy was needed to reach the transition of metamorphosis and puberty in the life cycle of the deep-sea bivalve *X. atlantica* compared to that of the remaining marine bivalve species, but the amount of energy needed was not substantially different from that needed by *T. navalis* (Table 2). The striking difference was for the energy threshold at puberty (${E}_{H}^{p}$), where 1000-fold less energy was needed for *X. atlantica* individuals than for the 29 other marine bivalves to reach this stage. If we scaled each data point to body size, then there were no differences in the energy needed to reach metamorphosis (${E}_{H}^{j}$) compared to that needed for the same range of body sizes (Fig. 2e); however, for puberty, the value (${E}_{H}^{p}$) of *X. atlantica* was the lowest compared to those of the other marine bivalve species (Fig. 2f).

**Table 2:** Comparison of primary and core parameters of the abj-DEB model of the Atlantic woodeater *Xylonora atlantica* with those of *Teredo navalis* at T ref = 293 K (https://www.bio.vu.nl/thb/deb/deblab/add_my_pet/entries_web/Teredo_navalis/Teredo_navalis_res.html) and the mean values of 29 marine bivalve species that are found in the AMP database (https://www.bio.vu.nl/thb/deb/deblab/add_my_pet/species_list.html), where abj-DEB models were used.

DEB parameters	*Xylonora atlantica*	*Teredo navalis*	Mean (SD) of 29 marine bivalves
Allocation fraction to soma ($\kappa$)	0.73	0.98	0.84 (0.18)
Maximum assimilation rate (${\left\{{\dot{p}}_{Am}\right\}}_{b}$)	14.36 J.cm^−2^.d^−1^	3.95 J.cm^−2^.d^−1^	14.68 (16.82) J.cm^−2^.d^−1^
Maximum assimilation rate after metamorphosis (${\left\{{\dot{p}}_{Am}\right\}}_{j}$)	38.77 J.cm^−2^.d^−1^	108.02 J.cm^−2^.d^−1^	73.15 (81.37) J.cm^−2^.d^−1^
Energy conductance at birth (${\dot{v}}_{b}$)	0.0053 cm.d^−1^	0.0025 cm.d^−1^	0.022 (0.015) cm.d^−1^
Energy conductance after metamorphosis $( {\dot{v}}_{j}$)	0.0143 cm.d^−1^	0.0652 cm.d^−1^	0.158 (0.233) cm.d^−1^
Reserve capacity $\left[{\mathrm{E}}_{m}\right]=\left\{{\dot{p}}_{Am}\right\}/\dot{v}$	2709 J. cm^−3^	1595 J. cm^−3^	667 (7972) J. cm^−3^
Volume specific costs for structure ($\left[{\mathrm{E}}_{G}\right]$)	2349 J.cm^−3^	2356 J.cm^−3^	2362 (28.7) J.cm^−3^
Volume specific somatic maintenance ($\left[{\dot{p}}_{M}\right]$)	29.22 J.cm^−3^.d^−1^	32.99 J.cm^−3^.d^−1^	21.62 (25.7) J.cm^−3^.d^−1^
Somatic maintenance rate coefficient (${\dot{k}}_{M}$)	0.012 d^−1^	0.014 d^−1^	0.009 (0.01) d^−1^
Maximum structural body length (*L*_*m*_ = Kappa {p_*Am*_}j/[P_M_])	0.358 cm	0.117 cm	1.13 (1.7) cm
Maturation threshold at birth (${E}_{H}^{b}$)	1.04e^−3^ J	3.5e^−6^ J	7e^−4^ (2e^−3^) J
Maturation threshold after metamorphosis (${E}_{H}^{j}$)	2.03e^−2^ J	6.32e^−2^ J	7.8e^−1^ (3.12) J
Maturation threshold at puberty (${E}_{H}^{p}$)	1.65 J	4.7 J	3108.74 (9300) J

*SD* standard deviation.

### Inferring functional traits from DEB

**Figure 3:**
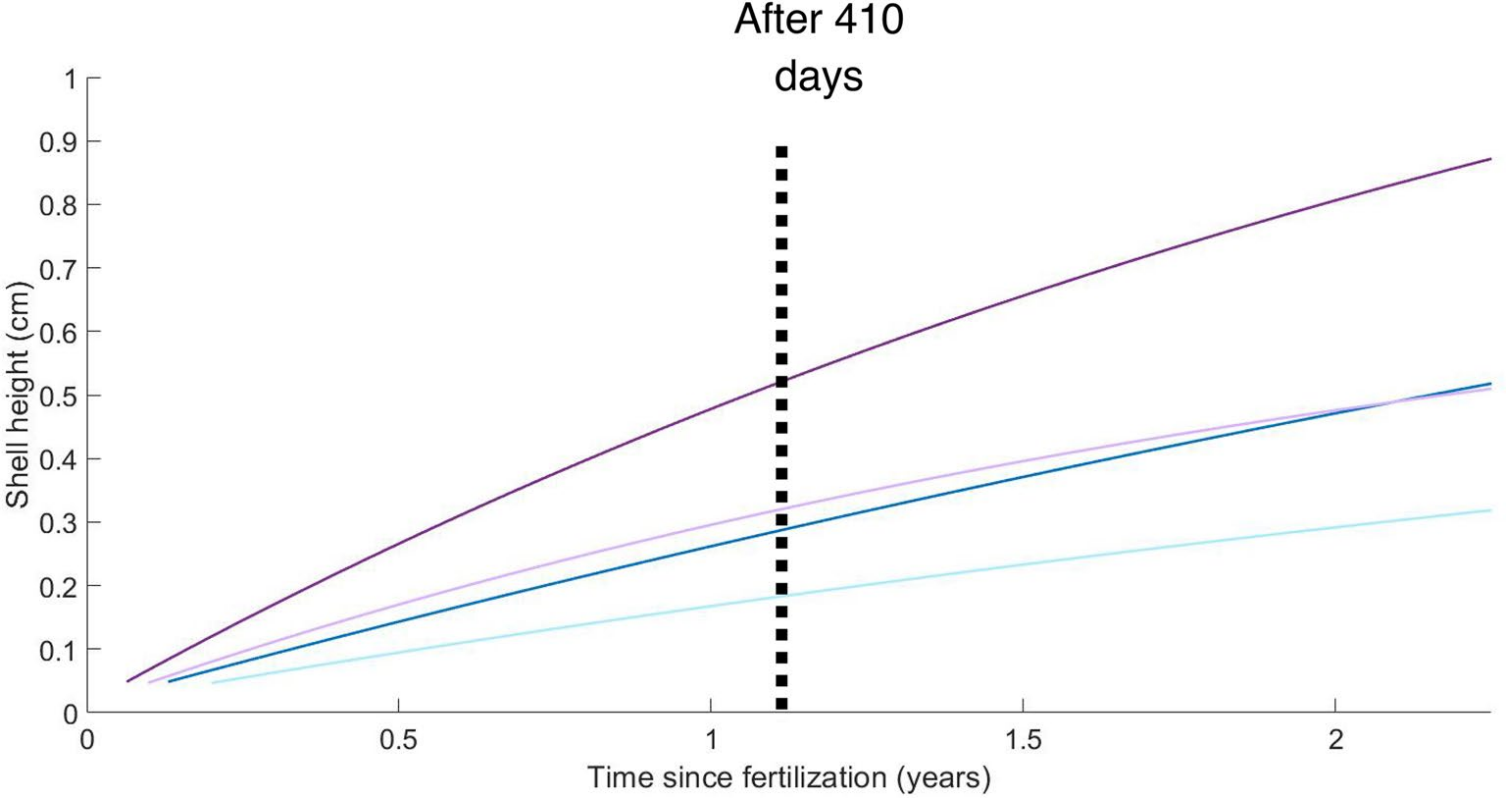
Prediction of the abj-DEB model of the evolution of the shell height of *Xylonora atlantica* under different environmental scenarios. At 2300 m in depth (T = 4 °C) with food level *f* = 0.5 (light blue) and *f* = 1 (dark blue); at 100 m in depth (T = 11 °C) with *f* = 0.5 (light purple) and *f* = 1 (dark purple) up to the lifespan at 4 °C (821 days).

Four environmental conditions were chosen, with a constant temperature (two modalities with 4 °C simulating the deep-sea environmental conditions and 11 °C simulating shallower water conditions such as at 100 m in depth in the North Atlantic Ocean) and constant food availability (two modalities with *f* = 0.5 and *f* = 1) (Fig. 3). For *f* = 0.5 (from fertilization), shell height (SH) reached ~ 0.25 cm at 4 °C and ~ 0.5 cm at 11 °C (Fig. 3) after 821 days (lifespan at 4 °C). For *f* = 1 (from fertilization), shell height reached ~ 0.5 cm at 4 °C and ~ 0.9 cm at 11 °C (Fig. 3). The mean SH of the *X. atlantica* individuals (excluding the dwarf males) that colonized CHEMECOLI in the colonization experiments at 2300 m after 410 days of deployment measured ~ 0.27 cm (SD = 0.05 cm, *n* = 333; Supplementary Fig. S2 online). Using the previous simulation of the growth of *X. atlantica* after more than a year, at *f* = 0.5 and T = 4 °C, the SH of *X. atlantica* reached only ~ 0.13 cm after 410 days (Fig. 3), while at *f* = 1 and T = 4 °C, the SH of the wood borers reached ~ 0.28 cm (Fig. 3). The results of the abj-DEB model matched the observations obtained in situ (Supplementary Fig. S2 online), suggesting that food level *f* was close to 1 in the colonization experiment after 410 days.

The von Bertalanffy growth rate was twofold higher at T = 11 °C than at T = 4 °C for the same *f*. *X. atlantica* took 316 days to reach puberty under the environmental conditions at 2300 m in depth at satiety compared with only 155 days under the environmental conditions at 100 m in depth (Table 3). For *f* = 0.5, age at puberty was only reached after 494 days under deep-sea conditions (Table 3). The total reproductive output (TRO) is generally low both at 4 °C (deep-sea conditions) and at 11 °C (100 m conditions) at satiety (Table 3). If food density is reduced by half at the deep-sea temperature, then the TRO is even smaller (1228 oocytes) (Table 3).

**Table 3:** Predictions of several fundamental functional traits of *Xylonora atlantica* using the abj-DEB model under different environmental scenarios of food level (*f*) and temperature (T, °C) with lifespans predicted by the abj-DEB model (~ 821 days at 4 °C and ~ 404 days at 11 °C).

*f* food level	1		0.5	
Temperature in °C	4	11	4	11
von Bertalanffy growth rate (d^−1^)	4.7e^−4^	9.5e^−4^	6e^−4^	12e^−4^
Lmax (maximum body size) (cm)	0.52	0.52	0.30	0.30
Age at puberty (in days) (1.9 mm)	316	155	494	243
Total reproductive output (TRO) in #	5026	5312	1228	1307

^#^Represents number of oocytes, *f* food level, *d* days.

The parameters of the abj-DEB model allowed us to estimate the time spent in the water column (pelagic larval duration: PLD) between hatching (trochophore) and settling (postlarval stage), giving a range of PLD from a minimum of 17 days (*f* = 1 at 16 °C; surface dispersal) to a maximum of 127 days (*f* = 0.5 at 4 °C; demersal dispersal) (Table 4). With demersal dispersal, PLD varied between ~ 3 months (*f* = 1) and ~ 4.5 months (*f* = 0.5). At depths of 100 m, PLD ranged from ~ 1 month (*f* = 1) to ~ 2 months (*f* = 0.5). With surface dispersal, PLD varied between 17 and 29 days.

**Table 4:** Predictions of the different larval stages and settlement ages according to different environmental scenarios in the North Atlantic Ocean.

*f* food level	0.5			0.8			1		
Temperature in °C	4	11	16	4	11	16	4	11	16
Age at hatching	5.6	5.6	5.6	5.6	5.6	5.6	5.6	5.6	5.6
Age at birth	38.4	18.8	12	36	17.7	10.2	35	17.2	10.6
Age after metamorphosis	139	68.2	42	109	53.7	32	99.6	48.9	30
Estimation of the PLD	127	56	29	96	41	19	87	36	17

The temperature at hatching was one of the in situ temperatures (4 °C). PLD represents pelagic larval duration, which encompasses the time between the trochophore stage (hatching) and the settlement of the pediveliger stage. We postulated that it took at least one week to complete the metamorphosis period from the pediveliger stage. Values are in days.

### *Xylonora atlantica* dwarf males in the deep sea: how is it possible?

Different scenarios were investigated to explain how some individuals (males) of *X. atlantica* reach sexual maturity with very slow or null growth of their shell. Either a decrease in the scaled functional response *f* or a change in the allocation fraction to soma ($\kappa )$ were tested. The mean shell height of the dwarf males recovered from CHEMECOLI after 410 days of deployment was 547.6 µm (SD = 31.5 µm; *n* = 64). First, at a temperature of 4 °C using the abj-DEB model, only a *f* value of 0.05 after the completion of metamorphosis gave a maximum shell height of ~ 0.055 cm after 410 days of deployment (Supplementary Figure S3a online). However, these individuals remained juveniles, as no energy was invested in the reproduction buffer to produce gametes (Supplementary Figure S3b online).

**Figure 4:**
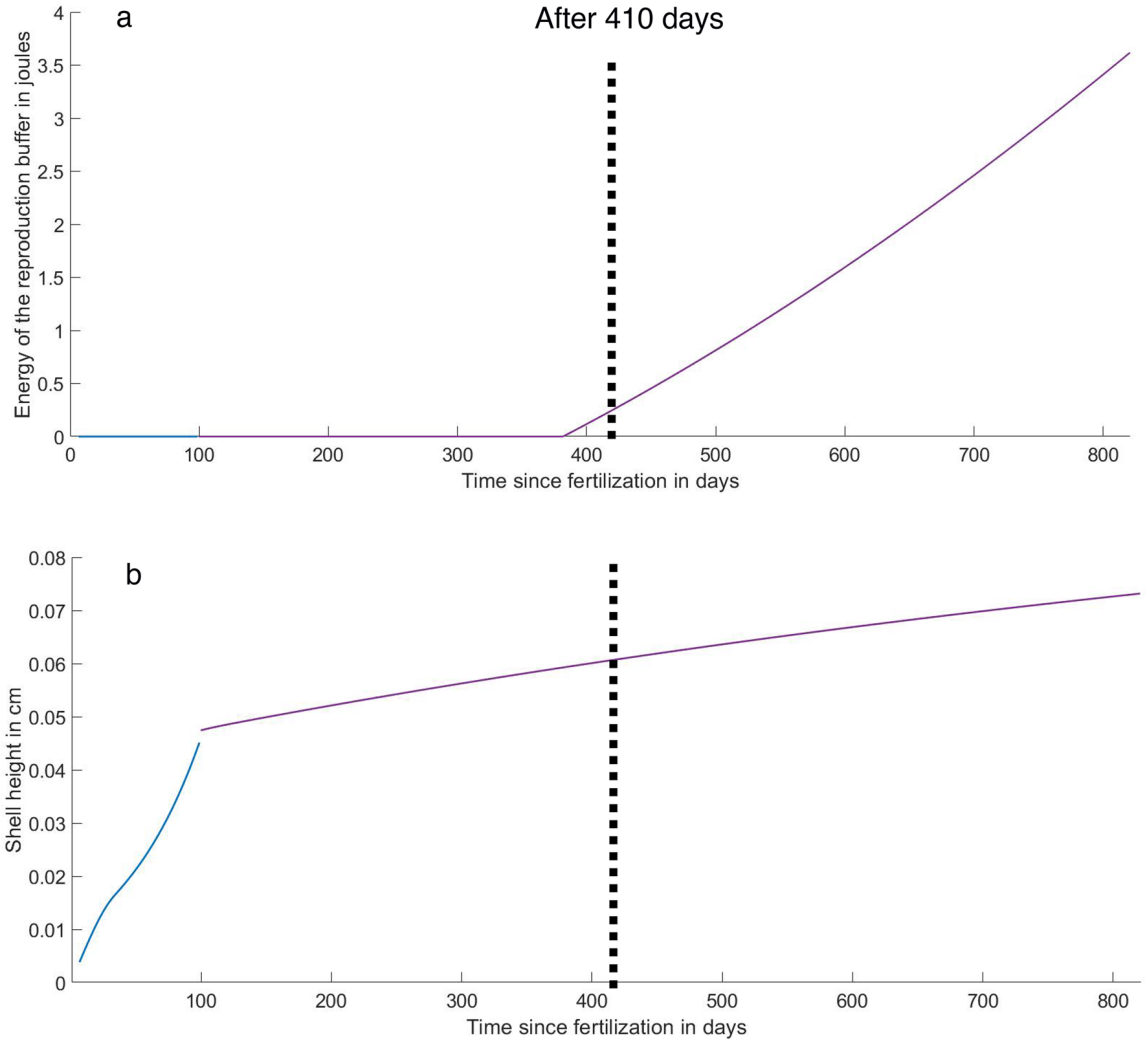
Prediction of the abj-DEB model of the evolution of (**a**) the shell height of dwarf male *Xylonora atlantica* at 2300 m in depth by changing $\kappa$ to 0.06 with *f* = 0.8 while settling (in purple). (**b**) Change (in purple) in the energy stored in the reproduction buffer at T = 4 °C and *f* = 0.8 after metamorphosis (in blue).

Second, by changing the value of $\kappa$ from 0.73 to 0.06 and fixing the *f* value at 0.8 after the completion of metamorphosis (‘juvenile’ stage), resulting in two $\kappa$ values during the life cycle of the dwarf male *X. atlantica*, the effect of dwarfism was well predicted. The new abj-DEB model allows xylophagaid individuals to reach a shell height of ~ 0.05/0.06 cm (Fig. 4a), similar to that observed in dwarf males, at 410 days with enough energy in the reproduction buffer to produce gametes (Fig. 4b). The growth of the dwarf males did not cease in the simulation (Fig. 4a), but growth was very slow, and at the end of the lifespan (821 days at 4 °C), the maximum shell height only reached 0.07 cm.

## Discussion

The first goal of the study was to evaluate whether the primary and core parameter values of the abj-DEB model of *Xylonora atlantica* were in line with those of shallow marine bivalves and more specifically with those of a shallow wood borer, such as *Teredo navalis*, for which the abj-DEB models were developed (AMP database^32^). A number of DEB parameters were in the same range, but some of them were very different and could highlight some specific adaptations to the deep-sea habitat or to the wood-fall habitat. For instance, the maximum assimilation rate $\left\{{\dot{p}}_{Am}\right\}$ of *X. atlantica* was twofold lower than those of shallower marine bivalve species but ten- to 15-fold higher than that of the symbiotic shallow marine bivalve *Thyasira* cf. *gouldi* that relies on food both from its sulfur-oxidizing bacteria and POM^33^. $\left\{{\dot{p}}_{Am}\right\}$ is linked to maximal structural body size (*L*_*m*_), where *X. atlantica* had a greater *L*_*m*_ than that of the thiotrophic bivalve *T.* cf. *gouldi*. However, *T. navalis* had a similar *L*_*m*_ to that of the symbiotic thyasirid, although *T. navalis* had a very high maximum assimilation rate. This difference probably arises from the way the two bivalves extract food. As a wood eater, *T. navalis* stores bacterial endosymbionts within its gills to synthesize enzymes that breakdown refractory material wood into sugars. This demonstrates the efficacy of cellulolytic bacteria and explains the growing interest in biotechnology arising from these new natural enzymes to produce biofuel^15^. The energy conductance ($\dot{v}$) was lower for the deep-sea wood borer *X. atlantica* and the shallow wood borer *T. navalis* than for the other shallow marine bivalve species (even the shallow symbiotrophic bivalves such as *T. flexuosa* or *T. cf. gouldi*, AMP database). This result reveals a lower mobilization capacity of reserve *E* in wood borers, enhancing a greater maximum reserve capacity ([E_m_]) as they are inversely proportional. Due to these higher [E_m_] values, in comparison to other marine bivalves, wood-boring bivalves may have higher survival rates in starvation periods, highlighting some specific adaptation to wood-fall habitats, which are usually fragmented and ephemeral in the ocean. This scenario would explain why some deep-sea xylophagaid species have been shown to survive in habitats other than wood (cable, plastic, vinyl, etc.) using these latter items only as shelters^30^. The volume-specific somatic maintenance $\left[{\dot{p}}_{M}\right]$ and the somatic maintenance rate coefficient (${\dot{k}}_{M}$) of *X. atlantica* that play a role in the turnover of the structure (DNA, enzymes, etc.) were much higher than those of the other marine shallower bivalve species (e.g., *T.* cf. *gouldi*, *Arctica islandica*, *M. edulis*, and *Cerastoderma edule*; AMP collections). Both values were not that different than those of *T. navalis* that lives in shallow water, revealing that living in the deep sea does not necessarily cost more than living in shallow water, as commonly believed, due to pressure^34^. The rate of activity of some enzymes may decrease due to high pressure, whereas others may be activated by high pressure^35^.

Less energy (2000-fold and 1000-fold) is needed to reach the transition stage of puberty ($${E}_{H}^{p})$$ in *Xylonora atlantica* and *Teredo navalis,* respectively compared to that of other shallow marine species and even for the same range of body sizes (*L*_*m*,_ maximum structural length). This result indicates some kind of adaptation in both juvenile wood borers that causes them to rush to produce small offspring in poor food and ephemeral environments. These results are consistent with the idea that there is low energy availability in wood-fall habitats, leading to a low maximum assimilation rate $$\left\{{\dot{p}}_{Am}\right\}$$ and enhancing the low energy threshold at each stage of the life cycle of endemic wood-fall species. The deep sea has long been seen as an energy-depleted ecosystem where at the abyssal seafloor, the carbon flux represents only 1% of the surface production^28^. Concomitantly, wood-fall communities can produce their own carbon flux by developing bacterial communities^36^ and metazoan communities^6^, such as those seen at hydrothermal vents^37, 38^, via chemoautotrophic or heterotrophic pathways producing local POM. However, the energy availability from wood-fall habitats, even considered oases in the deep sea^6^, may not be as productive as other chemosynthetic ecosystems, such as hydrothermal vents. The threshold for energy at birth ($${E}_{H}^{b}$$) of *X. atlantica* was similar to that of other marine bivalves and occurred while veligers were dispersing in the water column, supporting pelagic dispersal rather than local retention in wood-fall habitats. This may not be the case for *T. navalis,* where larvae are incubated in a female gill chamber for a certain period of time before being released^39^. The threshold for energy at birth in *T. navalis* was 1000-fold less than that in *X. atlantica*.

The general pattern of a decrease in fecundity and subsequently in total reproductive output (TRO) have been reported in different deep-sea phyla likely due to less favourable conditions such as low energy availability^40, 41^. TRO has an important role in population connectivity and ecosystem resilience^17^. In this study, by using modelling, the TRO of *X. atlantica* was low for an opportunistic species (TRO = 5026 oocytes at 2300 m deep for *f* = 1). Interestingly, *X. atlantica* was seen as an opportunistic species based on its production of millions of tiny eggs^8^. This paradoxical mismatch of low fecundity and high recruitment rates for deep-sea opportunistic species was discovered for deep-sea cocculinid limpets colonizing vascular plants^42^. Previous authors have hypothesized that this trait could result from natural selection or reflect phylogenetic constraints but may corroborate Thorson’s hypothesis^43^ that predation rates of larvae could be much lower at greater depths than at shallower depths. In the laboratory, some *X. atlantica* veligers could delay the completion of their metamorphosis^21^. This scenario would explain how these wood-boring bivalves can survive in ephemeral wood-fall habitats that are fragmented in the deep sea despite having a low fecundity and subsequently a weak number of offspring but with a greater larval survival rate than those of shallower marine species.

Deep-sea benthic species generally have low growth rates and long lifespans due to low temperatures and low energy availability. Metazoan species with a greater lifespan have been recorded for black corals of the family Antipatharia, with a lifespan of millennia (up to 3000 years)^44^. In chemosynthetic ecosystems, such as hydrothermal vents and cold seeps, where the energy availability is high compared to that in the surrounding deep sea area^45, 46^, the growth rates of chemosynthetic taxa (e.g., *Bathymodiolus thermophilus*, *Calyptogena magnifica*, and *Riftia pachyptila*) were shown to be in the same range of those of shallower species (a few cm per year)^47–49^. However, for other deep-sea bivalves from nonchemosynthetic ecosystems, such as the deep-sea protobranch bivalves, growth rates were shown to be much lower, with a range of 1 to a few mm per year^50^. Deep-sea bivalves are generally small and slow-growing^51^. Deep-sea xylophagaid wood-boring bivalve growth rates were most similar to those of other deep-sea bivalves from nonchemosynthetic ecosystems, despite being endemics to a wood-fall habitat, which is a chemosynthetic habitat^6^. A series of growth rates from several xylophagaid species were reported^24^, calculated from the colonization experiment deployment duration. Growth rates varied from 1 to 25 mm per year, and variations were due to species, depth ranges, wood type and size, and temperature. The highest growth rate was seen for xylophagaid species in the Mediterranean Sea^52^, where even at 1200 m in depth, the temperature reached 13 °C. Here, the abj-DEB model highlighted a von Bertalanffy growth rate of *Xylonora atlantica* that was twofold higher at 11 °C (~ 4.5 mm year^−1^) than at 4 °C (~ 2.3 mm year^−1^).

Earlier colonization experiments demonstrated that *X. atlantica* grows faster on pine than on oak^22^. Here, using an abj-DEB model, we confirm previous observations that growth is better on pine than on oak, as the *f* (scale functional response) calculated by the abj-DEB model gave a lower value than that of the pine substrate. For both temperatures (4° and 11 °C), the growth rate was lower at *f* = 0.5 compared to at *f* = 1 (satiety), inducing a lower maximum body size. The influence of temperature and primary productivity (as a proxy of food density) were tested on the growth rates of marine bivalves^53^, and temperature was shown to be a better predictor than food supply but only explained 10% of the variation. A significant relationship between an increase in the lifespan and a decrease in the von Bertalanffy growth rate with latitude has been demonstrated in marine bivalves^54^. The abj-DEB model developed for *X. atlantica* followed the general concept of the DEB theory that the decrease in temperature in this study linked with an increase in bathymetry induced a decrease in the von Bertalanffy growth rate and an increase in the lifespan.

Age at puberty (sexual maturation) is a significant trait that shows how long it takes for a species to reach a minimal size and the time to produce gametes. In this study, under deep-sea conditions (4 °C) and food density at satiety, it took 316 days to reach puberty, whereas at 100 m, it took only 155 days at the same food level. To date, only the age at puberty of *Xylophaga depalmai* for a wood-boring bivalve was recorded for a deep-sea wood-fall habitat^55^. But it was only the age since the start of the colonization experiment from the pediveliger stage (50 days, at 10–13 °C) that was reported, without taking into account the actual age of the xylophagaid since the fertilization period. The advantage of the DEB theory^16^ compared to other theories, such as the metabolic theory in ecology (MTE)^56^, is that the DEB theory takes into consideration the nutritional history of an organism at different life stages in its life cycle, and the DEB framework makes a priori predictions about broad scale patterns of many life-history traits between species^57^. Therefore, the abj-DEB model has allowed an exploration of different scenarios of the conditions under which *Xylonora atlantica* larvae may have experienced larval dispersal, deciphering surface, pelagic or demersal dispersal. In this study, several scenarios were simulated, resulting in different ranges of PLD. With demersal dispersal (4 °C), PLD ranged between 3 and 4.5 months and was similar to that estimated for the small chemosymbiotic bivalve *Idas modiolaeformis*, an inhabitant of both cold seeps and wood-fall habitats^58^. If dispersal of *X. atlantica* larvae occurred at the sea surface of the Atlantic Ocean, then the PLD calculated by the abj-DEB model was shorter (17–29 days) and fell exactly into the range of the PLD of shallow-water marine species^59^. One sample of a late *X. atlantica* umboveliger was thought to be recovered in a plankton net at the surface of Cape Cod Bay^21^. It seems that there is a trade-off for planktotrophic larvae to migrate vertically to increase food supply, but at the same time, the predation pressure may increase; in addition, demersal dispersal may permit a lower predation pressure but diminish the food supply. Data on the PLD of *X. atlantica* in this study can be used in connectivity studies with biophysical models to understand how metapopulations are connected, which is relevant for conservation purposes^59^. To date, only a few studies have developed biophysical models on deep-sea marine invertebrates^60–62^, as key life-history traits are scarce and physical data in the deep sea are still not easily accessible. An international effort is under way (Global Ocean Observing Strategy: www.goosocean.org) to deploy deep-sea moorings to obtain current data and other essential ocean variables (EOVs)^63^. These data could be used by physicists and in collaboration with biologists in further studies to develop biophysical models of deep-sea species to understand the connectivity and resilience of deep-sea populations. Here, the abj-DEB model could help estimate the growth of larvae according to the temperature and food conditions that the offspring will encounter during their dispersal. PLD could vary greatly, which would impact the different scenarios developed by any biophysical model.

As mentioned before, deep-sea habitats may be depleted in food supply, enhancing miniaturization of taxa due to drastic food constraints, while the gigantism observed in hydrothermal vents or cold seeps may be attributed to the decrease in predation, a pattern that is common in insular fauna^64^. Dwarf males of *Xylonora atlantica* with early postlarval stages that carry mature spermatozoids (therefore adults) were recovered in colonization experiments at a depth of 2300 m on the dorsal side of the reproductive female shell after 410 days of deployment^23,24^. This dwarfism was hypothesized to occur due to the increase in *X. atlantica* density within the colonization device, reducing the availability of wood both for shelter and food supply^23^. At the same time, stable isotopic analyses on the tissues of the dwarf males highlighted consumption of POM rather than of wood^24^. According to the present study, food availability (wood) was still high for ‘normal’ xylophagous male and female *X. atlantica* individuals after 410 days in the colonization experiment deployed on the mid-Atlantic ridge. However, the dwarf male *X. atlantica* had very slow growth but enough energy to be able to reproduce at the age of 410 days. This dwarfism strategy may have allowed more individuals per unit of area within the colonization device, enlarging the population size of *X. atlantica*, with dwarf males being mature, increasing the reproductive fitness of the xylophagaid population. By using the abj-DEB model developed for ‘normal’ *X. atlantica* individuals, it was impossible to provide enough energy to reproduce dwarf males despite decreasing the food level to a low density (*f* = 0.05) to obtain the size of a dwarf male. It was only by changing the allocation fraction to soma (*κ*) after the completion of metamorphosis that a good prediction of growth and reproduction was successfully obtained for dwarf males. Dwarf male growth of the shell did not cease after completion of metamorphosis but was very slow, with a fraction of the mobilized energy (*κ* ${\dot{p}}_{c}$) going mainly to somatic maintenance and the other fraction of energy (1- *κ*) ${\dot{p}}_{c}$ going to the reproduction buffer to produce spermatozoids and maturity maintenance. A change in *κ* during the life cycle of a species has been reported for the pond snail *Lymnaea stagnalis* due to a change in photoperiod conditions (i.e., LD16:8), switching the energy allocation in favour of reproduction at the expense of growth and its maintenance^65^. A second study^66^ on the maturation and growth of an amphibian, *Crinia georgiana,* described a change in *κ* value that decreased linearly from *κ*_*1*_ = 0.86 to *κ*_*2*_ = 0.61 between the ‘hatching’ and the ‘birth’ stages to obtain more energy into maturity at the expense of growth and its maintenance. The environmental trigger hypothesized in this latter study was the water level of the pond or the fact that the pond was ephemeral. This trigger was seen as a selective pressure. This latter example is in line with what is occurring in our study within the wood-colonization device, where wood cubes were ephemeral; second, the space available within the wood cubes for new colonists had reduced after a certain period of deployment and could have been a selective pressure on *κ*.

Another factor that could have induced dwarfism in the *Xylonora atlantica* population could be linked to Island’s rule, where within CHEMECOLI (the colonization device), exclusion of predators was permitted by the use of a plastic net of 2 mm mesh^24^. By selection, this scenario could have induced a reduction in body size to increase a high reproductive rate in a population such as seen on an island causing a dwarfing of mammals^56^. A regression analysis was conducted on the body size of deep-sea gastropods compared to their shallow-water relatives, highlighting that gigantism or miniaturization observed in deep-sea taxa could be a reminiscent pattern of insular faunas^64^. Small body size and early puberty characterize individuals with *r*-strategies^67^. *X. atlantica* is considered an opportunistic species^8^. However, its growth rate was slow (low energy conductance), which did not mirror an *r*-strategy. Furthermore, the maximization of individual density by miniaturization of the last recruits tends toward the *K*-strategy to maximize the carrying capacity^67^. Here, in the case of *X. atlantica,* the decrease in size was linked to the scarcity of wood substrate after several months of colonization, decreasing the ecological niche habitats (rather than food supply) but still providing enough energy to produce offspring to compensate for losses. Therefore, the life strategy of this deep-sea, wood-boring bivalve from the wood-fall habitat may be considered to have strategies between the *r* and *K* strategies.

## Conclusion

A DEB model conducted for a deep-sea benthic species (*Xylonora atlantica*) was developed successfully for the first time, revealing specific adaptations to deep-sea and wood-fall habitats, although this species has traits in common with shallow water marine bivalves. A series of functional traits (age at puberty, lifespan, total reproductive output, maximum body size and larval pelagic duration) according to food availability and temperature (with subsequent bathymetry) were deciphered using the abj-DEB model. These traits are scarce based on the fundamental knowledge of the deep sea, and in the next decade, we recommend that the DEB theory be developed for a greater number of deep-sea species to obtain more information on traits that are necessary for understanding the connectivity and resilience of species populations. However, real data on life-history traits and experimental data are still necessary to parameterize a DEB model on deep-sea fauna. DEB modelling will not preclude the continuation of sampling fauna in the deep sea but will expand knowledge on the traits of a targeted species.

## Methods

### Life cycle of *Xylonora atlantica* and the use of the abj-DEB model

Here, an abj-DEB model^32^ that differs from a standard model by having an extra juvenile life stage was applied to the Atlantic woodeater. This extra life stage takes place between the first feeding of the umboveliger larva (birth, ‘*b*’) and the end of its metamorphosis (‘*j*’), where metabolic acceleration (*s*_*M*_) occurs, resulting in exponential growth of the individual^68^ (Supplementary Material S1 online). During the dispersal phase in the water column, the onset of feeding is triggered when the maturity threshold at birth (*b*) is reached ($Eh$ ≥ ${E}_{H}^{b}$). The energy of the reproductive flux (Supplementary Material S1 online) occurs only to increase maturity with the increase in complexity. The digestive system of umboveliger larvae is functional, where particle organic matter (POM) is acquired through its velum (feeding apparatus). During the dispersal phase, while planktotrophy occurs, the larval shell, prodissochonch II (PDII), increases in size, reaching its maximum length at the settlement stage, and larvae are considered isomorphic with a constant shape coefficient (*δ*_*Me*_). The umboveliger, while settling on wood debris, loses its velum, developing a foot that becomes a pediveliger. When metamorphosis is completed, new anatomical features appear similar to those of adults, such as a new visible dissoconch shell that hosts specialized features, and, rasping denticles, allowing a young juvenile wood borer to switch its diet to xylophagy. At this later stage, the juvenile xylophagous species is considered isomorphic with a constant shape coefficient (*δ*_*M*_), and growth follows a von Bertalanffy growth curve where energy flux still continues from maturation until puberty ${E}_{H}^{p}$ (Supplementary Material S1 online). Finally, individuals reach puberty, with males producing spermatogonia and females producing oogonia from initial germ cells. At this stage, the energy of the maturity branch is used only for maturity maintenance and storage in the reproduction buffer, *E*_*R*_ (Supplementary Fig. S1 online).

### Synthesis of data for *Xylonora atlantica* and DEB parameter value estimation

For all zerovariate data, the value of the scaled functional response *f* was set to 1, considering that only the ‘best individual’ was used. Temperature was taken into account in the zerovariate dataset for age and in all the univariate dataset with rates in order to apply a temperature correction (Supplementary Material S1 online).

A number of early-stage zerovariate data (Supplementary Table S2 online) were obtained from a larval culture^21^ where some mature female *X. atlantica* released spawned eggs spontaneously in the laboratory, revealing internal fertilization. Larvae were reared until the pediveliger stages. Several key data were used, such as age and length at hatching (trochophore), birth (umboveliger) and metamorphosis (pediveliger). For puberty (occurrence of sexual maturity) and fecundity, a histological study^24^ was used to provide shell height (SH) and wet weight of the youngest mature female *X. atlantica* and the maximum reproduction rate. The age at puberty was deduced from a colonization experiment^22^ carried out at 100 m in the North Atlantic Ocean giving shell height following time and the size at first reproduction defined by the previous histological study^24^. Only one study^8^ has reported the maximum shell height and lifespan observed in *X. atlantica*.

The univariate dataset (Supplementary Table S2 online) was retrieved both from the literature and from the authors. First, two in situ colonization experiments^22^ at 100 m in depth followed the shell height (SH) of *Xylonora atlantica* recruits for almost one year at the edge of the continental shelf, south of Cape Cod (North Atlantic Ocean), using two different types of substrates (pine and oak). Second, specimens (333 juveniles/adults *X. atlantica*) from a colonization experiment (CHEMECOLI)^23, 24^ carried out at 2300 m in depth in the mid-Atlantic ridge for one year and fixed in FISH fixative were used. The shell height (SH) of each individual was measured using a BA210 Motic digital compound microscope with Motic Image 3.0 software and weighed (wet weight) to calculate the shape (*δ*_*M*_) for juveniles/adults. The scaled functional response *f* was set to 1 for the pine colonization experiments, whereas the other univariate dataset remained free, as for the oak colonization experiments.

Parameter estimation^32^ of the DEB model was carried out using the *Xylonora atlantica* dataset of online Supplementary Table S2. The estimation of the parameters was possible using the package DEBtool, which is updated periodically (https://github.com/add-my-pet/DEBtool_M) with MATLAB R2020a software using an abj-DEB model. The goodness of fit^32^ was estimated by two criteria in the parameter estimation procedure: first, the mean relative error (MRE) ranges from 0 (predictions match data perfectly) to infinity (no match), and second, the symmetric mean square error (SMSE) varies from 0 (predictions match data perfectly) to 1 (no match).

### Inferring functional traits of deep-sea species

The best fit of the abj-DEB model of *Xylonora atlantica* was used to follow the evolution of the compartments of reserve (*E*), structure (*V*) and reproduction buffer (*E*_*R*_) from fertilization over time according to the equations in Supplementary Material S1 online. Four environmental conditions were chosen, with one simulating deep-sea environmental conditions (4 °C) at two food levels (*f* = 0.5 and 1) and a second simulating shallower water conditions at 100 m in depth (11 °C) at two food levels (*f* = 0.5 and 1). The values *V* over time were then converted into physical shell height (maximum body size) with the equations from Supplementary Material S1 online.

Several other functional traits, such as the von Bertalanffy growth rate, the total reproductive output (TRO) equal to the total number of oocytes accumulated during the lifespan of the species and the age at first sexual maturation (puberty), were estimated under the two scenarios of temperature (4 °C and 11 °C) and food levels (*f* = 0.5 and 1). The abj-DEB model for *Xylonora atlantica* was used to reconstruct the chronology of the early-life stages of the population of *X. atlantica* recovered in the CHEMECOLI experiments^23, 24^ at 2300 m in depth on the mid-Atlantic ridge at 4 °C. Several scenarios were envisioned: one with demersal pelagic larval dispersal at 2300 m in depth (4 °C); a second scenario with sea surface larval pelagic dispersal using a mean annual temperature value of 16 °C for the Atlantic Ocean; and a third scenario with pelagic larval dispersal at 100 m in depth (11 °C). All these scenarios were performed from the umboveliger stage (birth) to the settlement stage (metamorphosis), in which *f* values varied from 0.5 to 0.8 to 1 but with an initial stage (embryo) fixed at *f* = 1 and T = 4 °C (spawning from females *X. atlantica* at the deep-sea wood-fall at 2300 m).

### Dwarf male in *Xylonora atlantica* in the deep sea: how is it possible?

By manipulating the value of $\kappa$ (Supplementary material S1 online) and the scaled functional response (*f*) manually, we provided a possible explanation for the growth delay of an individual male *X. atlantica* that retains a young juvenile body size but acquires enough energy in its reproduction buffer to produce male gametes (far less than the normal size at sexual maturity). The shell heights of 64 dwarf male *X. atlantica* sampled and fixed in FISH fixative^23^ were measured using a BA210 Motic digital compound microscope with Motic Image 3.0 software.

## Supplementary Information

Supplementary Information.

## Data Availability

The abj-DEB model of *Xylonora atlantica* is accessible on the database of Add-My-Pet (https://www.bio.vu.nl/thb/deb/deblab/add_my_pet/entries_web/Xylonora_atlantica/Xylonora_atlantica_res.html) and provides the results output of the model. Several scripts in Matlab were used to infer the functional traits of *X. atlantica* and can be requested to SMG.
