# Supplementary material for: Inferring functional traits in a deep-sea wood-boring bivalve using dynamic energy budget theory
Source: Sci Rep. 2021 Nov 22;11:22720. doi: 10.1038/s41598-021-02243-w (PMC8608800; doi:10.1038/s41598-021-02243-w)
Supplement: Supplementary file 1 — Supplementary Information. [file 41598_2021_2243_MOESM1_ESM.pdf]

Inferring functional traits in a deep-sea wood-boring bivalve using dynamic energy budget theory

Gaudron, S.M.[1,2,*], Lefebvre, S.[1,+], Marques, G.M.[3,+]

[1]UMR 8187 Laboratoire d'Océanologie et de Géosciences (LOG), Université de Lille, ULCO, CNRS, F-59000 Lille, France.
[2]Sorbonne Université, UFR 927, F-75005 Paris, France.
[3] MARETEC - Marine, Environment & Technology Center, LARSyS, Instituto Superior Técnico, Universidade de Lisboa, Lisboa, Portugal

*Email (corresponding author): sylvie.gaudron@sorbonne-universite.fr
[+]These authors contributed equally to this work

Supplementary Material S1. Description of the Dynamic Energy Budget (DEB) model.

Dynamic Energy Budget (DEB) describes the energy flows within an individual between three compartments (state variables) in the organism: the reserve (E), the structure (V) and the reproduction buffer (E_R) and a state variable, maturity (E_H), that determines its life stage: embryo, juvenile or adult[1] (Fig. S1). The standard DEB model considers an isomorphic organism assuming that food assimilation rate is dependent on surface area (i.e. structural volume to the power 2/3) and maintenance is proportional to structural volume. During the embryo stage, there is no assimilation, and the individual uses its reserve inherited from the mother. During this stage and the juvenile stage, a fraction (1-κ) of the mobilized flux is dedicated to maturity and its maintenance. During the adult stage, the fraction (1-κ) is dedicated to reproduction and maturity maintenance of the level reached at puberty. For a constant food density, the standard DEB model follows the so-called von Bertalanffy growth model. The calibration of a set of 12 primary parameters offers the description of the full-life cycle dynamics and life-history traits using a specific estimation method[2]. DEB theory predicts many types of intra- and interspecific scaling relationships, and offers a sound theoretical basis of energy budget compared to the metabolic theory in ecology [3]. A DEB model consists in a system of three ordinary differential equations following first order dynamics. Assimilated substrate first enters a reserve pool (E) which is then mobilized to fuel two pathways following the κ-rule: a fixed κ fraction is allocated to perform growth of the structural volume (V) and its maintenance and the remaining fraction (1-κ) is available for maturity (i.e. increase of complexity, E_H, and its maintenance) and reproduction (E_R)[3]. The three ordinary differential equations use a combination of seven different fluxes (Table S1) [1]. Temperature corrections are made to the rates considered by the model in the equation of fluxes (e.g. the surface-area specific maximum assimilation rate, $\{\dot{p}_{Am}\}$ J.cm^{-2}.d^{-1}), the energy conductance, $\dot{v}$ (cm.d^{-1}), the specific volume-linked somatic maintenance rate, $[\dot{p}_M]$ (J.cm^{-3}.d^{-1}), and the maturity maintenance rate coefficient, $\dot{k}_J$ (d^{-1}), see Table S1). Rates are temperature corrected using

$$\dot{k}(T) = \dot{k}_1 exp\left(\frac{T_A}{T_{ref}} - \frac{T_A}{T}\right),$$

where T_A is the Arrhenius temperature (K), $\dot{k}_1$the rate of interest at the reference temperature T_{ref} and $\dot{k}$ the rate of interest at temperature T.
The assimilation rate $\dot{p}_A$ is proportional to the surface area

$$\dot{p}_A = \{\dot{p}_{Am}\}\ s_M f L^2$$

with parameters explained in the Table S1. The scaled functional response f is a Michaelis-Menten function called also the Holling type II functional response. This is a limiting function that varies between 0 (i.e. starvation) and 1 (i.e. satiety) depending on the substrate concentration (i.e. food availability).

$$f = \frac{X}{X + X_K}$$

with X the concentration of food and X_K the half saturation constant. Within the typified DEB models, the *abj* type was used. The *abj* model differs from the standard model by having an extra juvenile life stage between birth (*b*) and metamorphosis (*j*). During this period, a metabolic acceleration[4] is considered, which is found to happen in many species that have a larval phase, frequently coinciding with morphological metamorphosis[2], and resulting in an exponential growth of the organism between birth and metamorphosis. The links between observable metrics (physical length and wet weight) and the DEB model quantities are made with the shape coefficient δ (varying between $\delta = \delta_{Me}$ for embryo and $\delta = \delta_M$ after metamorphosis), the density of wet structure d_V ($g.cm^{-3}$) of wet reserve d_E ($g.cm^{-3}$) and of dry reserve d_{Ed} ($g.cm^{-3}$), the specific chemical potential of reserve μ_{Ed} ($J.Cmol^{-1}$ of reserve), and the molar weight of reserve w_{Ed} ($g.Cmol^{-1}$) (Table S1). Here, we assume that $d_V = d_E = 1$ $g.cm^{-3}$, $d_{Ed} = 0.16$ $g.cm^{-3}$, $\mu_{Ed} = 550\,000$ $J.Cmol^{-1}$ and that $w_{Ed} = 23.9$ $g.Cmol^{-1}$.

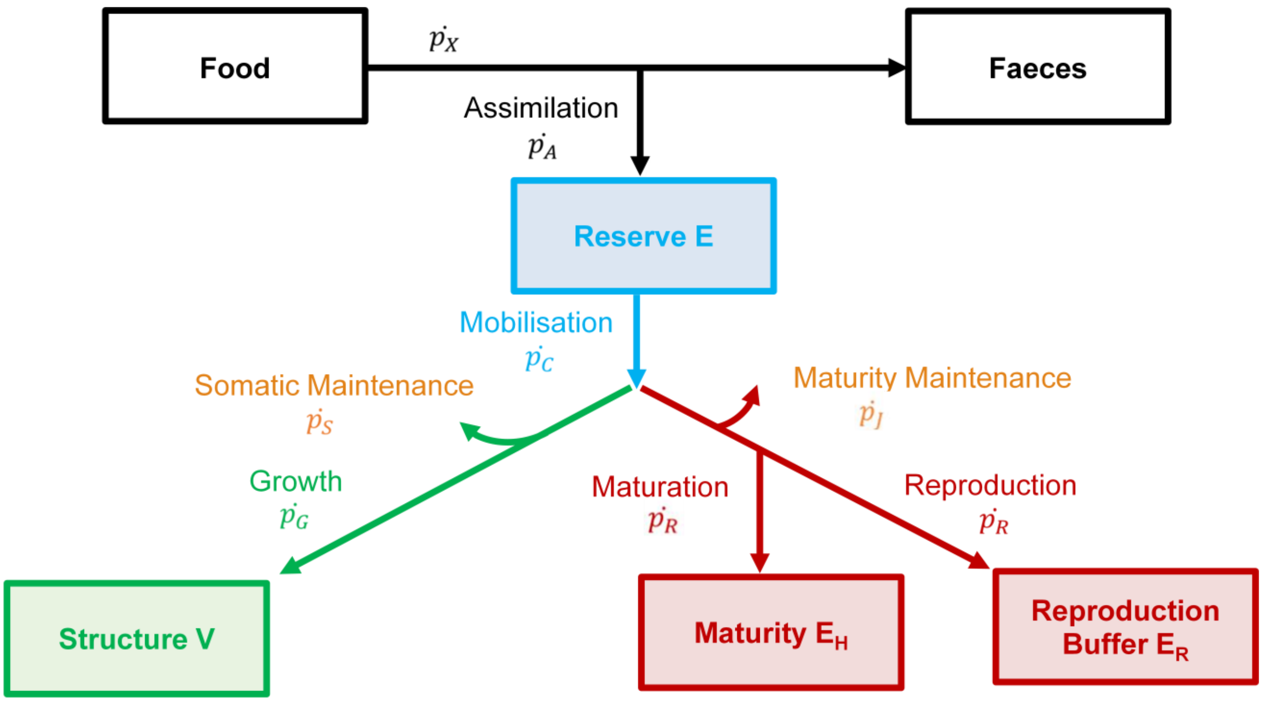

Supplementary Figure S1. Schematic representation of DEB model but modified from its initial origin[5]. Boxes are the state variables with (1) E, the reserve; (2) V, the structure; (3) the energy invested either in Maturity, E_H or in reproduction buffer, E_R. All arrows are energy flows and are detailed in Table S1.

Supplementary Table S1: State variables, fluxes, metric relationships, acceleration and shape coefficient of the *abj*-DEB model and associated mathematical expressions[1,3,4,6]. L is the structural length (cm) with $L = V^{1/3}$ and L_b and L_j are the structural lengths at birth and metamorphosis respectively. d_V is the density of wet structure, d_E the density of wet reserve, d_{Ed} the density of dry reserve, μ_{Ed} the specific chemical potential of reserve and w_{Ed} the molar weight of dry reserve. L_w is the physical total length at time t of the individual. W_w is the wet weight at time t of the individual. W_d is the dry weight at time t of the individual.

State variables	Reserve	$\frac{dE}{dt} = \dot{p}_A - \dot{p}_C$
	Structure	$\frac{dV}{dt} = \frac{\dot{p}_G}{[E_G]}$
	Maturity	$if\ E_H < E_H^p\ \frac{dE_H}{dt} = \dot{p}_R else\ \frac{dE_H}{dt} = 0$
	Allocation to reproduction	$if\ E_H \geq E_H^p\ \frac{dE_R}{dt} = \kappa_R\,\dot{p}_R\ else\ \frac{dE_R}{dt} = 0$
Fluxes	Ingestion	$\dot{p}_X = \frac{p_A}{\kappa_X}$
	Assimilation	$\dot{p}_A = \{\dot{p}_{Am}\} s_M f V^{2/3}$
	Mobilisation	$\dot{p}_C = E\,\frac{\dot{v} s_M V^{2/3}[E_G] + \dot{p}_S}{\kappa E + V[E_G]}$
	Somatic maintenance costs	$\dot{p}_S = [\dot{p}_M]V$
	Maturity maintenance costs	$\dot{p}_J = \dot{k}_J E_H$
	Growth	$\dot{p}_G = \kappa\dot{p}_C - \dot{p}_S$
	Reproduction and Maturity	$\dot{p}_R = (1-\kappa)\dot{p}_C - \dot{p}_J$
Metric relationships	Physical length (cm)	$L_w = L/\delta_M$
	Wet weight (g)	$W_w = d_V L^3 + E\,\frac{d_E}{\rho_E d_{Ed}}$
	Dry weight (g)	$W_d = d_{Vd} L^3 + \frac{E}{\rho_E}$
Acceleration coefficient	$if\ E_H < E_H^b\ s_M = 1\ \ if\ E_H^b \leq E_H < E_H^j\ \ s_M = L/L_b\ else\ s_M = L_j/L_b\ \ if\ E_H \geq E_H^j$	
Shape coefficient	$if\ E_H < E_H^b\ \delta = \delta_{Me}\ if\ E_H^b \leq E_H < E_H^j\ \delta = \delta_{Me} + (\delta_M - \delta_{Me})\left(\frac{L - L_b}{L_j - L_b}\right) else\ \delta = \delta_M\ if\ E_H \geq E_H^j$	

Supplementary Table S2. Data used in the *abj*-DEB parameters estimation for *Xylonora atlantica*.

Type of data	Data	Abj-DEB notation	References
Zerovariate	Age at hatching (trochophore)	a_h	[7]
	Age at birth (first feeding) (umboveliger)	a_b	[7]
	Time since birth until metamorphosis (until the juvenile stage)	t_j	[7]
	Time since birth to puberty	t_p	[8]
	Lifespan	a_m	[9]
	Height of the trochophore	Lw_h	[7]
	Shell height of the umboveliger	Lw_b	[7]
	Shell height at the metamorphosis	Lw_j	[7]
	Shell height at puberty	Lw_p	Gaudron unpublished
	Wet weight at puberty	W_{wp}	Gaudron unpublished
	Total maximum shell height	L_{wi}	[9]
	Maximum reproduction rate	R_i	[10]
Univariate	Time -Total Length (2 substrates)	tL_1, tL_2	[8]
	Total Length –Wet Weight	LW	Gaudron unpublished

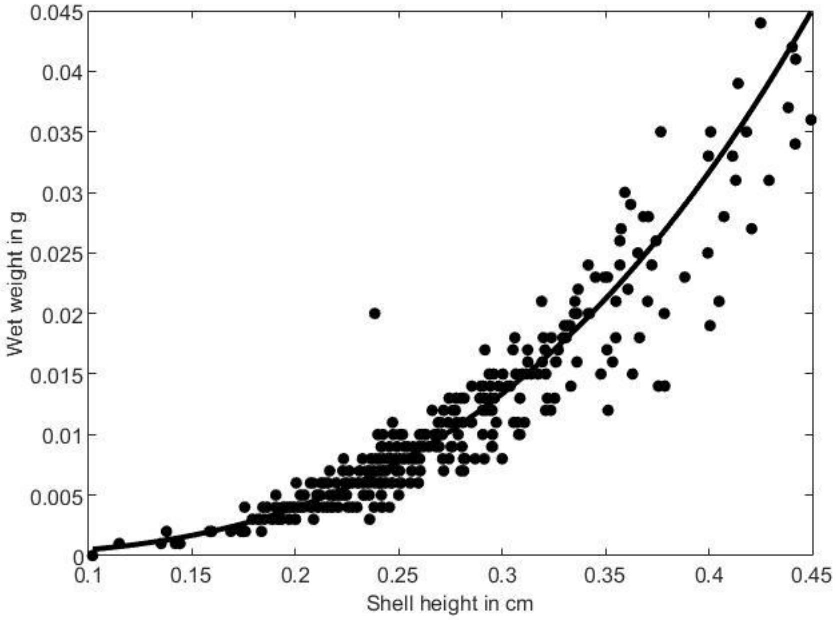

Supplementary Figure S2. Data (dots) and predictions (lines) of the wet weight as a function of shell height from juvenile to adult *Xylonora atlantica* individuals (this study) using an abj-DEB model. The corresponding value of the shape (δ_M) was 0.599 with a relative error of 0.16.

Supplementary Table S3. Synthesis of the primary and auxiliary parameters provided by the parameterization of the abj-DEB model for *Xylonora atlantica*. The values of $\dot{h}_a$ was taken from the generalized animal[1] and s_M is given for a scaled functional response of 1 after metamorphosis and is the ratio of the structural lengths *Lj* to *Lb*.

Parameter	Symbol	Value from abj-DEB model	Unit
Reference temperature	T_{ref}	293.15	K
Fraction of food energy fixed in reserve	κ_X	0.8	-
Arrhenius temperature fixed	T_A	8000	K
Energy conductance	$\dot{v}_b(\dot{v}_j)$	5.3^{-3} ($1.4e^{-2}$)	$cm.d^{-1}$
Allocation fraction to soma	κ	0.73	-
Reproduction fraction fixed in eggs	κ_R	0.95	-
Volume specific costs of structure	$[E_G]$	2349	$J.cm^{-3}$
Maturation threshold for hatching	E_H^h	$7.5e^{-6}$	J
Maturation threshold for birth	E_H^b	$1.04e^{-3}$	J
Maturation threshold for metamorphosis	E_H^j	$2.04e^{-2}$	J
Maturation threshold for puberty	E_H^p	1.65	J
Weibull ageing acceleration	$\dot{h}_a$	$5.9e^{-5}$	d^{-2}
Gompertz stress coefficient	s_G	$1.00e^{-4}$	-
Acceleration rate	s_M	2.7	-
Maximum assimilation rate	$\{\dot{p}_{Am}\}$	14.36	$J.cm^{-2}.d^{-1}$
Specific somatic maintenance rate	$[\dot{p}_M]$	29.22	$J.cm^{-3}.d^{-1}$
Maturity maintenance rate	$\dot{k}_j$	0.002	d^{-1}
Specific density of wet structure and reserve	$d_V = d_E$	1	$g.cm^{-3}$
Specific density of dry structure and reserve	$d_{Vd} = d_{Ed}$	1	$g.cm^{-3}$
Shape juvenile/adult	δ_M	0.5994	-
Shape larve	δ_{Me}	0.629	-
Zoom factor	z	0.3582	-

Supplementary Figure S3. Prediction of the abj-DEB model of the evolution of **(a)** a young juvenile *Xylonora atlantica* shell height that grew very slowly its dissoconch shell and **(b)** its energy allocated to reproduction being null at T = 4°C with f = 0.05 at juvenile stage.

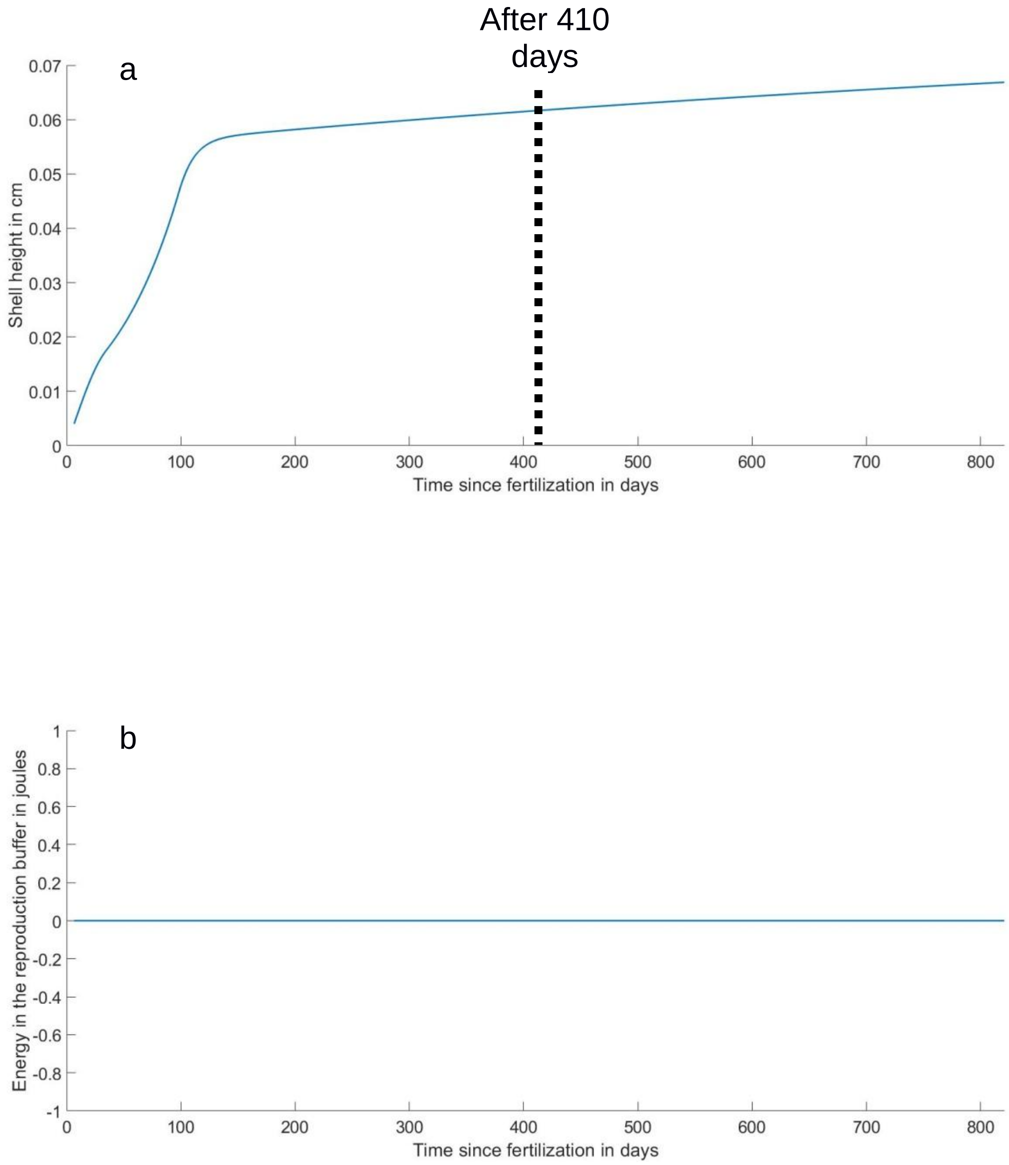

References

1. Kooijman, S. A. L. M. Dynamic energy budget theory for metabolic organisation. Cambridge University Press (2010).

2. Lika, K. *et al.* The 'covariation method' for estimating the parameters of the standard Dynamic Energy Budget model I: philosophy and approach. *J. Sea Res.* **66**, 270–277, https://doi.org/10.1016/j.seares.2011.07.010 (2011).

3. Van der Meer, J. An introduction to Dynamic Energy Budget (DEB) models with special emphasis on parameter estimation. *J. Sea Res.* **56**, 85–102, https://doi.org/10.1016/j.seares.2006.03.001 (2006).

4. Kooijman, S. A. L. M. Metabolic acceleration in animal ontogeny: An evolutionary perspective. *J. Sea Res.* 94, 128–137, https://doi.org/10.1016/j.seares.2014.06.005 (2014).

5. Marques, G. M. *et al.* The AmP project: comparing species on the basis of dynamic energy budget parameters. *PLoS Comput. Biol.* **14**, 1–23, https://doi.org/10.1371/journal.pcbi.1006100 (2018).

6. De Cubber, L., Lefebvre, S., Lancelot, T., Denis, L. & Gaudron, S. M. Annelid polychaetes experience metabolic acceleration as other Lophotrochozoans: Inferences on the life cycle of Arenicola marina with a Dynamic Energy Budget model. *Ecol. Modell.* 411, 108773, https://doi.org/10.1016/j.ecolmodel.2019.108773 (2019).

7. Kooijman, S. A. L. M. & Lika, K. Resource allocation to reproduction in animals. *Biol. Rev.* **89**, 849–859, https://doi.org/10.1111/brv.12082 (2014).

8. Culliney, J. L. & Turner, R. D. Larval development of the deep-water wood boring bivalve, *Xylophaga atlantica* Richards (Mollusca, bivalvia, pholadidae). *Ophelia* **15**, 149–161, https://doi.org/10.1080/00785326.1976.10425455 (1976).

9. Romey, W., Bullock, R. & Dealteris, J. Rapid growth of a deep-sea wood-boring bivalve. *Cont Shelf Res* **14**, 1349–1359 (1994).

10. Turner, R. Wood-boring bivalves, opportunistic species in the deep sea. *Science* **180**, 1377–1379, https://doi.org/10.1126/science.180.4093.1377 (1973).

11. Gaudron, S. M., Haga, T., Wang, H., Laming, S. R. & Duperron, S. Plasticity in reproduction and nutrition in wood-boring bivalves (*Xylophaga atlantica*) from the Mid-Atlantic Ridge. *Mar. Biol.* **163**, 1–12, https://doi.org/10.1007/s00227-016-2988-6 (2016).
